# Knowledge, attitude, and status of nitrite inhalant use among men who have sex with men in Tianjin, China

**DOI:** 10.1186/s12889-017-4696-7

**Published:** 2017-09-04

**Authors:** Zheng Zhang, Li Zhang, Feng Zhou, Zhen Li, Jie Yang

**Affiliations:** 1 Chaoyang Center for Diseases Control and Prevention, Beijing, 100021 China; 2 Beijing Center for Diseases Prevention and Control, Beijing Research Center of Preventive Medicine, Room 205, No.16 Hepingli Middle Street, Beijing, 100013 China; 3 School of Public Health, Capital Medical University, Beijing, 100069 China; 4 Tianjin Deep Blue Volunteers Workgroup, Tianjin, 300131 China

## Abstract

**Background:**

Nitrite inhalants have become popular as recreational drugs among the homosexual population in some developed countries since the 1980s. These drugs, also called RUSH in China, have become attractive among Chinese men who have sex with men (MSM) in the past few years. The aim of this cross-sectional study was to understand the knowledge, attitude, and status of nitrite inhalant use among Chinese MSM.

**Methods:**

The study participants were recruited from Tianjin, China between April and August 2012. Information, including demographics, sexual behavior, and RUSH use, was obtained through structured interviewer questionnaires. Blood samples were also collected to identify the status of HIV, HSV, and syphilis infections.

**Results:**

A total of 500 participants were interviewed. Of the participants, 64.0% knew that RUSH could increase sexual pleasure and 38.6% of the participants had used RUSH at least once. The mean duration of RUSH use was 1.5 years. Among the participants who were familiar with RUSH, 60.0% had heard of RUSH for the first time after 2011, 55% received information about RUSH via the internet, and only 42.2% knew the side effects of RUSH. RUSH users were more likely to work in companies (Odds ratio [OR]: 2.61; 95% CI: 1.65–4.12), live with homosexual partners (OR: 1.88; 95% CI: 1.19–2.92), not live alone (OR: 2.26; 95% CI: 1.29–3.96), smoke cigarettes (OR: 1.49; 95% CI: 1.02–2.17), use alcohol (OR: 1.63; 95% CI: 1.12–2.39), and seek sexual partners on the internet (OR: 2.59; 95% CI: 1.50–4.50).

**Conclusions:**

The impact of RUSH abuse on the expanding HIV epidemic among MSM has been demonstrated in China. Our findings suggest that the communication and awareness of health hazard of recreational drugs should be reinforced in HIV prevention education, especially through new media. Future research is needed to further explore how integrative strategies should be used to reduce the substance abuse and risky sexual behaviors.

## Background

In the past 30 years, nitrite inhalants, including amyl, butyl, and isobutyl nitrites, have become popular as recreational drugs among the homosexual population in developed countries [1–3]. Amyl nitrites have a long history of abuse and are commonly referred to as “poppers.” Butyl and isobutyl nitrites have become attractive substitutes when amyl nitrites are refrained from non-medical use [4]. Butyl and isobutyl nitrites can be easily obtained in some commercial applications, such as intermediates for perfumes and antifreeze preparations. Butyl and isobutyl nitrites are usually given suggestive trade names, such as “Aroma of Men,” “RUSH,” and “Highball.” These products have been reported to have effects on facilitating and enhancing sexual intercourse and are extensively used by men who have sex with men (MSM) [1]. Along with the acquired immunodeficiency disease (AIDS) epidemic, studies have shown a correlation between nitrite inhalant use and unsafe sexual behaviors, which lead to human immunodeficiency virus (HIV) infection and increased risk of HIV seroconversion among MSM [5–13].

There are 654,000 people living with HIV (PLHIV) in China and 27.5% of the PLHIV were MSM as of September 2016 [14]. Injection drug use was the initial route of HIV transmission, but the risk of sharing needles, syringes, or other sources of bloodborne infections has decreased significantly over the past few years because of the national needle exchange program. In a previous study, we found that injection drug use is very rare in the MSM population in China [15]. Other studies in China in the past few years revealed the prevalence of some new drugs, such as methamphetamine, ketamine, and methylenedioxyamphetamine, instead of opium and heroin [16, 17]. RUSH use has become popular during recent years. Studies from the United States showed that the prevalence of popper use varied from 25% to 35% since the initiation of highly active antiretroviral therapy [12, 18–21]. A cohort study in Canada found a similar nitrite use ratio, which was 34% in 1995–1996, and remained unchanged in 2002–2004 [3]. In Japan, a national internet survey of popper use was conducted in 2003. The results showed a very high prevalence of 63.2% [2]; however, recently, more and more Chinese MSM communities asked us to pay close attention to the use of nitrite inhalants and their potential health effects. A cross-sectional study carried out among HIV-positive MSM in Shanghai in 2010 suggested that the risk of HIV transmission increased three times when RUSH was used in unsafe sexual intercourse [22]. Another cross-sectional survey conducted among MSM in Beijing in 2012 reported that the HIV prevalence was 9.0% among nitrite inhalants users and 3.3% among non-users [23]. A recent report from Nanjing showed the increasing trend of RUSH use from 2014 to 2015 among MSM. RUSH use was associated with HIV infection [24]. Moreover, previous research demonstrated that MSM who had ever used nitrite inhalants had behavior characteristic of preferring sexual pleasure, casual sexual partners, multiple sexual partners, and engaging in risky sexual behaviors [3, 21, 23, 25, 26]. According to these reports, nitrite inhalants or male perfumes can be easily purchased online in China. Most of the nitrite inhalant users in China are MSM. The aim of our current study was to explore the history and prevalence of nitrite inhalant use and to assess the user’s knowledge and attitude about RUSH among the MSM population in Tianjin, China.

## Methods

### Study design and participants

This cross-sectional study was conducted between April and August 2012. Study participants were recruited from Tianjin, China through the Tianjin Deep Blue Volunteers Workgroup (http://www.tjtz.org), a local non-government organization. The MSM community in Tianjin is one of the most competent communities in China, with much experience in HIV/AIDS prevention and control. Participants of our current study were recruited from this community using convenience sampling and three approaches were used. First, study materials were distributed through internet advertisement by the Tianjin Deep Blue Volunteers Workgroup. Second, peer educators distributed cards containing information that was relevant to our study at MSM-common locations, such as MSM clubs, bars, and bathhouses. Third, MSM individuals visiting the HIV Voluntary Consulting and Testing (VCT) Clinic, located at the Tianjin Deep Blue Volunteers Workgroup, were also introduced to the study and encouraged to refer other MSM to participate. The selection criteria for our study were males, age > 18 years, HIV-negative status or unknown by self-report, having had sex with other men in the past 6 months, and willingness to participate and provide written informed consent. Written informed consent was obtained before the interview and laboratory tests. Structured interviewer-administered questionnaires and blood samples were collected to identify the HIV, human simple virus (HSV)-2, and syphilis infection status. All interviewers were peer-educators from the Tianjin Deep Blue Volunteers Workgroup and all interviewers had HIV/AIDS health educator certification through the National Center for AIDS/STD Control and Prevention.

#### Data collection

Data from individual participants were collected in a private room using a questionnaire administered by a trained interviewer. A unique code was assigned to each participant to link the questionnaire and the specimens. Questionnaire items about socio-demographic characteristics were similar to the socio-demographic characteristics in a previous study [27]. Items about RUSH were developed according to the information known from the MSM communities and the results of previous studies carried out in developed countries. To determine the sequence of questions for the questionnaire, a pilot survey was conducted with 20 individuals recruited in the VCT clinic. Questionnaire items about RUSH included the date when the participant first heard of RUSH, the age at first use of RUSH, RUSH knowledge (i.e., the name and the characteristics of nitrite inhalant products, the application and attitude of RUSH, and the common effects of RUSH use), the commercial channels of getting RUSH, the frequency of RUSH use, the combined use of other drugs, if any, the side effects of using RUSH, the history of STDs and HIV infection, and the history of other health-related behaviors, such as drinking alcohol and smoking.

#### Sample collection and laboratory tests

Blood samples were collected for laboratory testing of HIV, HSV, and syphilis serology. HIV infection status was determined using an enzyme-linked immunosorbent assay (ELISA; Shanghai Kehua Bio-Engineering Co. Ltd., Shanghai, China) and confirmed with western blot (HIV Blot 2.2 WBTM, Genelabs Diagnostics, Singapore). Syphilis infection status was also determined by ELISA (Beijing Kinghawk Pharmaceutical Co. Ltd., Beijing, China) and confirmed with a passive particle agglutination test, which detects the antibodies against *Treponema pallidum* (TPPATM, FUJIREBIO, Japan). ELISAs were performed to measure the IgG antibodies against HSV-2 (Trinity Biotech, USA).

#### Statistical analysis

The questionnaire answers were double-entered and checked with the EpiData software (EpiData 3.02 for Windows; The Epi Data Association Odense, Denmark). After cleaning, the data were then converted and analyzed using the Statistical Analysis System (SAS 9.2 for Windows; SAS Institute Inc., Cary, NC, USA). The general information about RUSH knowledge and the prevalence of RUSH use were shown using descriptive analysis. Using univariate logistic regression analysis, we also compared the characteristics of demographics, sexual orientations, sexual partner-seeking patterns, diagnosed STDs, and the current statuses of HIV, syphilis, and HSV-2 infection between men who did and did not report the use of RUSH. The correlates were identified using odds ratios (ORs) with 95% confidence intervals (CIs).

## Results

### Demographic characteristics

A total of 500 participants were interviewed with informed consent. The age of the participants ranged from 18 to 68 years, with a mean age of 29.7 ± 8.1 years. Of the participants, 98.4% were of Han nationality and 52.2% had a junior college degree or higher. Of the participants, 86.0% were identified as homosexual, 14.0% as bisexual and other. Of all participants, 63.6% resided in Tianjin and 75.2% were single (Table 1). Antibody testing results showed that 38 (7.6%) and 43 (8.7%) participants were HIV seropositive and HSV-2 seropositive, respectively. Fifty-eight (11.6%) participants were infected by syphilis and 40 had symptoms of syphilis (Table 4).

**Table 1:** Baseline characteristics of study participants (*N* = 500)

Participant variables	Participants No. (%)
Age (years)	
≤ 20	31(6.2)
21–30	306 (61.2)
31–40	107 (21.4)
41–50	40 (8.0)
> 50	16 (3.2)
Han Ethnic	
No	8 (1.6)
Yes	492 (98.4)
Years of education	
≤ 12	239 (47.8)
> 12	261 (52.2)
Marital status	
Single	376 (75.2)
Married/ divorced/widowed	124 (24.8)
Monthly income (RMB)	
≤ 3000	390 (78.0)
> 3000	110 (22.0)
Registered residence	
Not	182 (36.4)
Yes	318 (63.6)
Occupation	
others	175 (35.0)
Students	53 (10.6)
Staff in company	151 (30.2)
Service	114 (22.8)
Live status	
alone	274 (54.8)
Only with homosexual partners	105 (21.0)
With homosexual or heterosexual partners	61 (12.2)
Only with heterosexual partners	59 (11.8)
Self-reported sexual orientation	
Bisexual, transgender, or uncertain	74 (14.0)
Homosexual	425 (86.0)

### Knowledge and attitude to RUSH

Among the 500 MSM study participants, 64.0% (320/500) knew that RUSH was an inhaled drug and could be used before or during sexual intercourse. Of the participants, 1.6% (5/320) heard of RUSH for the first time before 2005, 35.0% (112/320) between 2009 and 2010, and 60.0% (192/320) after 2011. The information which participants learned about RUSH came from various MSM activities. The internet was the main source of information for 55.0% (176/320) of participants, homosexual partners for 38.8% (124/320), friends for 36.9% (118/320), MSM-frequented venues (bathhouse, clubs, and bars) for 2.2% (7/320), and MSM parties for 1.6% (5/320). There was one participant who first heard of RUSH from his heterosexual partner. Of the participants, 49.4% had introduced RUSH to their friends or sexual partners. With respect to the attitude of the health effects of RUSH, 55.4% of the participants reported “did not know,” 12.0% reported “definitely had side effects,” 30.6% reported “probably have,” and 2.0% thought RUSH was safe. Of the participants, 45.9% (147/320) reported knowing that RUSH was a corrosive liquid, and 42.2% (134/320) were familiar with the side effects, such as dizziness (37.8%), severe palpitations (30.6%), shortness of breath (15.0%), blurred vision (12.2%), erectile dysfunction (6.6%), confusion (4.4%), tinnitus (4.1%), and vomiting (0.9%). (Table 2).

**Table 2:** Knowledge and attitude to RUSH among MSM in Tianjin (*N* = 500)

Content	Participants No. (%)
Knew RUSH	
No	180 (36.0)
Yes	320 (64.0)
Source of information about RUSH (*n* = 320)	
Internet	176 (55.0)
Homosexual partners	124 (38.8)
Friends	118 (36.9)
MSM-frequented venues (bathhouse, clubs and bars)	7 (2.2)
MSM party	5 (1.6)
Heterosexual partner	1 (0.3)
Frequency of RUSH use	
Never	307 (61.4)
Just one time	18 (3.6)
Rarely	22 (4.4)
Sometimes	54 (10.8)
Often	55 (11.0)
Every time	44 (8.8)
RUSH did harm to human’ health	
Did not know	277 (55.4)
Definitely	60 (12.0)
Probably	153 (30.6)
Safe	10 (2.0)
Symptom of the side effect of RUSH (*n* = 320)	
Dizziness	121 (37.8)
Severe palpitation	98 (30.6)
Breath hardness	48 (15.0)
Blurred vision	39 (12.2)
Erectile dysfunction	21 (6.6)
Confusion	14 (4.4)
Ttinnitus	13 (4.1)
Vomiting	3 (0.9)

### Status of RUSH use among MSM

In the present study, 193 (38.6%) participants reported that they had used RUSH at least once, and 67.4% (130/193) reported they used RUSH within 1 year. The histories of using RUSH varied from 0.1 to 12.0 years, with a mean history of 1.5 ± 1.1 years. The ages of the participants when first using RUSH varied from 17.1 to 61.6 years, with a mean age of 27.3 ± 7.0 years. Regarding the reasons of using RUSH, increased sexual pleasure accounted for 67.4% (130/193), required by a partner for 63.7% (123/193), pain relief in sexual intercourse for 33.7% (65/193), sexual stimulus for 17.1% (33/193), and drug dependence for 14.0% (27/193). Of the RUSH users, 49.2% (95/193) owned RUSH products, and 69.5% (66/95) of this group of respondents bought RUSH from an online shop, 35.8% (34/95) from friends, 7.4% (7/95) from overseas purchases, and 3.2% (3/95) from adult stores. Among the RUSH users, 22.8% (44/193) used RUSH during every sexual intercourse, 28.5% (55/193) used RUSH often, 28.0% (54/193) used RUSH sometimes, 11.4% (22/193) seldom used RUSH, and 9.3% (18/193) only tried RUSH once. Of the participants, 67.4% (130/193) reported that they had used RUSH to increase voluptus. Simultaneous use of RUSH with other illicit drugs was very rare (only 2 of the participants reported methamphetamine hydrochloride use and 2 declined to report). Of RUSH users, 80.3% reported considering use of condoms when they were using RUSH during sexual encounters, 98 (50.8%) RUSH users had recommended RUSH to his partners or friends, and 92.4% of these introducers thought at least one of their partners or friends began to use RUSH because of their recommendation.

Compared to men who did not report RUSH use, RUSH users were more likely to work in a company (OR: 2.61; 95% CI: 1.65–4.12), live with their homosexual partners (OR: 1.88; 95% CI: 1.19–2.92), live with their homosexual or heterosexual partners (OR: 2.26; 95% CI: 1.29–3.96), be gay (OR: 1.99; 95% CI: 1.14–3.47), smoke (OR: 1.49; 95% CI: 1.02–2.17), use alcohol (OR: 1.63; 95% CI: 1.12–2.39), and seek sexual partners on the internet (OR: 2.59; 95% CI: 1.50–4.50; Tables 3 and 4).

**Table 3:** Compare of characteristics of study participants, by self-reported use of RUSH (*N* = 500)

	Use of RUSH		
Factors	Yes No. (%)	No No. (%)	Odds Ratio (95% CI)
Age (years)			
≤ 20	10 (5.2)	21 (6.8)	1
21–30	122 (63.2)	184 (60.0)	1.39 (0.63, 3.06)
31–40	47 (24.4)	60 (19.5)	1.65 (0.71, 3.83)
41–50	11 (5.7)	29 (9.5)	0.80 (0.29, 2.22)
> 50	3 (1.5)	13 (4.2)	0.49 (0.11, 2.10)
Han Ethnic			
No	4 (2.1)	4 (1.3)	1
Yes	189 (97.9)	303 (98.7)	0.62 (0.15, 2.52)
Years of education			
≤ 12	88 (45.6)	151 (49.2)	1
> 12	105 (54.4)	156 (50.8)	1.16 (0.81, 1.67)
Marital status			
Single	159 (82.4)	217 (70.7)	1
Married/ divorced/widowed	34 (17.6)	90 (29.3)	0.51 (0.33, 0.80)
Monthly income (RMB)			
≤ 3000	146 (75.6)	244 (79.5)	1
> 3000	47 (24.4)	63 (20.5)	1.25 (0.82, 1.92)
Registered residence			
Not	67 (34.7)	115 (37.5)	1
Yes	126 (65.3)	192 (62.5)	1.14 (0.78, 1.65)
Occupation			
others (student, unemployed, retired)	49 (25.8)	126 (41.6)	1
Students	22 (11.6)	31 (10.2)	1.82 (0.96, 3.45)
Staff in company	76 (40.0)	75 (24.8)	2.61 (1.65, 4.12)
Service	43 (22.6)	71 (23.4)	1.56 (0.94, 2.57)
Live status			
alone	94 (49.0)	180 (58.6)	1
With homosexual partners only	52 (27.1)	53 (17.3)	1.88 (1.19, 2.92)
With homosexual or heterosexual partners	33 (17.2)	28 (9.1)	2.26 (1.29, 3.96)
With heterosexual partners only	13 (6.8)	46 (15.0)	0.54 (0.28, 1.05)

**Table 4:** Compare of behavior and disease prevalence of study participants, by self-reported use of RUSH (*N* = 500)

	Use of RUSH		
Factors	Yes No. (%)	No No. (%)	Odds Ratio (95% CI)
Self-reported sexual orientation			
Bisexual, transgender, or uncertain	19 (9.9)	55 (17.9)	1
Homosexual	173 (90.1)	252 (82.1)	1.99 (1.14, 3.47)
HIV-1 serostatus			
Negative	181 (94.3)	281(94.5)	1
Positive	12 (5.7)	26 (5.5)	0.72 (0.35, 1.46)
History of syphilis			
No	171 (88.6)	271 (88.3)	1
Yes	22 (11.4)	36 (11.7)	0.97 (0.55, 1.71)
HSV-2 serostatus			
Negative	178 (93.7))	273(89.8)	1
Positive	12 (6.3)	31 (10.2)	0.59 (0.30, 1.19)
Smoking			
No	115 (59.6)	211 (68.7)	1
Yes	78 (40.4)	96 (31.3)	1.49 (1.02, 2.17)
Alcohol use			
No	116 (60.1)	219 (71.3)	1
Yes	77 (39.9)	88 (28.7)	1.63 (1.12, 2.39)
Primary way of seeking sexual partners			
Park, public toilet and others^a^	19 (9.8)	63 (20.5)	1
Bars and clubs	3 (1.6)	6 (2.0)	1.66 (0.38, 7.27)
Bathhouse	2 (1.0)	22 (7.2)	0.30 (0.07, 1.40)
Internet	169 (87.6)	216 (20.3)	2.59 (1.50, 4.50)

^a^park, public toilet, bars, bathroom and club

## Discussion

To our knowledge, this is the first study that investigated the knowledge and attitude regarding nitrite inhalant use among the MSM population in China. Most of the participants first heard of RUSH after 2011. Along with its popular use among the MSM population, RUSH has raised new challenges to the health and behaviors of the general public in China. A recent study conducted in Kunming, China reported that there was no significant difference in RUSH use between homosexual and bisexual MSM [28]. This phenomenon indicated that RUSH use could be expanded to the general population.

Nitrite inhalant use has become very popular among the MSM population in Western countries. The prevalence of nitrite inhalant use was reported to be 36.3%–60% in Europe [29, 30] and approximately 35.0% in the United States [9, 16, 31]. Furthermore, The National LGB Drug and Alcohol Database identified that alcohol, poppers, and cannabis were the most commonly used MSM drugs [31]. In this study, the prevalence of RUSH use in the past 1 year was higher than the prevalence in Western countries, but lower than the prevalence in Asian countries or other parts of China [2, 3, 23, 32]. The ages of the participants ranged from 18 to 68 years. The mean age was 29.7 ± 8.1 years, which was similar to the previous MSM studies carried out in Beijing, China and in the United States [23, 31, 33]. The rate of RUSH use in the past 1 year in our survey (26%) was lower than the Beijing MSM study (47.3%) [23]. The difference in prevalence between the current study and the previous Beijing study may be explained by the differences of economic and cultural environments between the two regions. In comparison, Beijing is a more open and modernized city with a much larger internal migratory population than Tianjin [23]. According to the 2010 National Population Census data, the itinerant population accounted for 35.9% of permanent residents in Beijing, but only about 25% in Tianjin. The culture and environment is more diverse due to the large number of immigrants. In our study, 63.6% participants resided in Tianjin; however, the percentage was 31.8% in Beijing [23]. Therefore, the MSM population from Beijing might have more opportunities to communicate with exotic communities than MSM from Tianjin [34–37]. Although the prevalence of RUSH use in Tianjin was lower than other regions of China, the prevalence of RUSH use was still high when compared with data from Western countries [2, 20, 32, 38, 39].

Our study showed that MSM, who had consumed alcohol, smoked, had better jobs, or lived with homosexual partners and sought partners through the internet, were prone to RUSH use. These findings were consistent with other studies [10, 33, 40], especially those also carried out in China [23, 40]. MSM who had the habit of smoking or drinking alcohol might be more inclined to use stimulants. They might have a better job, which could lead to a better income and better access to the substances and substance information [23]. The characteristics of RUSH users and the source of information about RUSH in this study were also similar to other studies [2, 33, 40]. In light of these findings, preventive strategies based on our study could be popularized among MSM in China. Studies from the United States suggested nitrite inhalant use increased the risk of HIV or STI infection [6, 16, 20]. Studies conducted in other cities in China also noted the association of nitrite inhalant use and HIV infection [22–24]. Although we did not find any relationship between RUSH use and HIV infection, there may be regional differences and biases in the convenient sampling methods used in our study.

In the past decades, there have been numerous studies carried out to investigate the relationship between RUSH use and sexual behaviors and/or HIV infection status of the users. However, studies focusing on the level of knowledge of RUSH among MSM are very rare. Our results showed that the MSM population in China knew little about the side effects of RUSH. The lack of knowledge may directly lead to health hazards to the MSM individual users and could even lead to some serious social and public health issues. In light of these concerns, our study will provide some baseline information about the knowledge of and attitude towards recreational drug use among MSM population in China. Health education strategies should be developed to prevent the epidemic of RUSH use and the potential boost of HIV transmission among MSM communities in China. The information of the RUSH distribution channels also indicated that the transaction of RUSH was quite free and active in China.

Nevertheless, several limitations of this study should be kept in mind. First, potential bias about sensitive questions could not be excluded because the questionnaires were interviewer-administered. Second, the participants in our study might not represent the general MSM population due to the potential limitations of enrollment methods. Third, it should be kept in mind that the results of antibody tests might not represent the actual infection status of syphilis infection and the test results might also be influenced by the quality of the samples. Fourth, the cross-sectional study design also has its own limitations on the correlation analysis. Therefore, confirmation of these results is needed by further case-control studies or prospective studies with more sensitive testing methods. Although the data in this study was dated, our findings still gave baseline information on the prevalence of RUSH use and user knowledge among MSM population in Tianjin, China. We consider the surge of its popularity could negatively affect HIV prevention and control. The results of our study should benefit design strategies for appropriate health education.

## Conclusion

The impact of RUSH abuse on the expanding HIV epidemic among MSM has been demonstrated in China. Our findings suggest that the awareness of health hazard of recreational drugs should be highlighted in HIV preventive education, especially through new media. Future research is needed to further explore how integrative strategies should be used to reduce substance abuse and risky sexual behaviors.

## Abbreviations

AIDS: acquired immunodeficiency diseases
ELISA: enzyme-linked immunosorbent assay
HIV: human immunodeficiency virus
MSM: men who have sex with men
PLHIV: people living with HIV
VCT: voluntary consulting and testing
